# Arbeitsintensität – Stand und Trends: Querschnittlicher Datenvergleich auf Grundlage der BIBB-/BAuA-Erwerbstätigenbefragungen 2012, 2018 und 2024

**DOI:** 10.1007/s00103-026-04291-x

**Published:** 2026-09-07

**Authors:** Andrea Lohmann-Haislah, Katja Schuller, Wibke Leistner

**Affiliations:** Bundesanstalt für Arbeitsschutz und Arbeitsmedizin, Nöldnerstr. 40–42, 10317 Berlin, Deutschland

**Keywords:** Termin- oder Leistungsdruck, Multitasking, Leistungsgrenze, Veränderung der Arbeitsintensität, Arbeitswelt, Deadline or performance pressure, Multitasking, Working at the limit of one’s capacity, Changes in work intensity, Working environment

## Abstract

**Einleitung:**

Arbeitsintensität gilt als ein zentraler arbeitsbezogener Risikofaktor für die Gesundheit von Beschäftigten. Sie beschreibt das Verhältnis zwischen Arbeitsmenge und zur Verfügung stehender Zeit. Ihre seit den 1990er-Jahren zu verzeichnende Zunahme wird als ein zentrales Merkmal des Wandels der Arbeit angesehen.

**Methoden:**

Mittels der BIBB-/BAuA-Erwerbstätigenbefragung 2024 (BIBB Bundesinstitut für Berufsbildung; BAuA Bundesanstalt für Arbeitsschutz und Arbeitsmedizin) wird bei rund 18.000 abhängig Beschäftigten der Frage nach der aktuellen Verbreitung von Arbeitsintensität nachgegangen und mittels Daten aus den Jahren 2012 und 2018 untersucht, wie sie sich entwickelt hat. Dazu werden 3 Arbeitsintensitätsmerkmale herangezogen. Die Daten werden stratifiziert nach Geschlecht, Arbeitszeit, Qualifikationsniveau und beruflicher Tätigkeit betrachtet.

**Ergebnisse:**

Frauen, Vollzeitbeschäftigte sowie Beschäftigte mit höherem beruflichen Anforderungsniveau sind in stärkerem Maße von Arbeitsintensität betroffen als Männer, Teilzeitbeschäftigte oder Beschäftigte mit geringem beruflichen Anforderungsniveau. Insgesamt haben das Multitasking zu- und Termin- oder Leistungsdruck sowie das Arbeiten an der Leistungsgrenze abgenommen. Allerdings gelten diese Veränderungen nicht für alle Beschäftigtengruppen gleichermaßen. Nach wie vor gibt es Gruppen mit auffällig hoher sowie auch mit eher zunehmender Arbeitsintensität.

**Diskussion:**

Insbesondere für diese Beschäftigten müssen Bemühungen um gut gestaltete Arbeit verstärkt werden. Trotz positiver Gesamttendenzen muss Arbeitsintensität weiterhin als zentraler Schlüsselfaktor im Arbeitsschutz, in der Prävention und in der Arbeitsgestaltung im Blick behalten werden, denn langanhaltende hohe Arbeitsintensität trägt zur Entwicklung von Herz-Kreislauf- und Muskel-Skelett-Erkrankungen sowie depressiven Störungen bei.

**Zusatzmaterial online:**

Zusätzliche Informationen sind in der Online-Version dieses Artikels (https://doi.org/10.1007/s00103-026-04291-x) enthalten.

## Abstract (en)

**Introduction:**

Work intensity is considered a key work-related risk factor for employee health. It describes the ratio between the amount of work and the time available. Its increase since the 1990s is seen as a key feature of the changing nature of work.

**Methods:**

By using the 2024 BIBB/BAuA Employment Survey, the current prevalence of work intensity among around 18,000 employees was examined and compared with data from 2012 and 2018 to investigate how it has developed. Three work intensity characteristics were used for this purpose. The data were stratified according to gender, working hours, professional requirements, and occupational activity.

**Results:**

Women, full-time employees, and employees with higher professional requirements are more affected by work intensity than men, part-time employees, or employees with low job requirements. Overall, multitasking increased and deadline or performance pressure and working at the limit of one’s capacity decreased. However, these changes do not apply to all employee groups equally. There are still groups with noticeably high and even increasing work intensity.

**Discussion:**

Efforts to create well-designed work must be intensified, especially for these employees. Despite positive overall trends, work intensity must continue to be kept in mind as a key factor in occupational safety, prevention, and work design, because prolonged high work intensity contributes to the development of cardiovascular, musculoskeletal, and depressive disorders.

## Einleitung

Die Zunahme der Arbeitsintensität gilt bereits seit den 1990er-Jahren als eines der wesentlichen Kennzeichen des Wandels der Arbeit und als zentrales Merkmal im arbeitsbezogenen Stressgeschehen. Arbeitsintensität beschreibt das Verhältnis zwischen quantitativen Arbeitsanforderungen (z. B. Arbeitsmenge, Arbeitsgeschwindigkeit), qualitativen Arbeitsanforderungen (z. B. Schwere, Komplexität und Qualität) und der für die Bearbeitung der Arbeitsaufgabe zur Verfügung stehenden Zeit [1, 2]. Beschäftigte, die unter hoher Arbeitsintensität arbeiten, müssen z. B. häufig verschiedene Arbeiten bzw. Vorgänge gleichzeitig betreuen oder an der Grenze ihrer Leistungsfähigkeit arbeiten [3]. Reicht die zur Verfügung stehende Zeit für die Bearbeitung der Arbeitsaufgabe nicht aus, besteht das Risiko, dass die zur Bewältigung erforderliche Anstrengung und die damit verbundene Beanspruchung zu gesundheitlichen Beeinträchtigungen führen [4].

Eine hohe Arbeitsintensität äußert sich im Erleben der Beschäftigten unter anderem als Zeit- und Leistungsdruck, dem Beschäftigte häufig mit einer Ausdehnung ihrer Arbeit(szeit) in das Privatleben (Extensivierung) oder dem Versuch, schneller zu arbeiten oder mehrere Dinge gleichzeitig zu erledigen (Intensivierung), begegnen. Beide Strategien gehen ebenfalls mit gesundheitlichen Beeinträchtigungen einher [3]. Eine hohe Arbeitsintensität hängt nachweislich in Kombination mit geringen Handlungsspielräumen (*Job Strain*) mit der Entwicklung von Herz-Kreislauf-Erkrankungen, Muskel-Skelett-Erkrankungen und depressiven Störungen [5–8] zusammen. Aber auch die quantitativen Arbeitsanforderungen an sich sind ein zentraler arbeitsbezogener Risikofaktor für die Gesundheit der Beschäftigten [9]. Damit ist die Arbeitsintensität ein zentraler Schlüsselfaktor im Arbeits- und Gesundheitsschutz, dem eine herausragende Rolle für die präventive Arbeitsgestaltung zum Schutz der Beschäftigten vor Gefährdungen durch psychische Belastung zukommt [10–12].

Die Ursachen für eine Intensivierung der Arbeit sind vielfältig und resultieren einerseits aus gesellschaftlichen, technologischen sowie wirtschaftlichen Entwicklungen und andererseits aus betrieblichen Strategien, um auf diese zu reagieren. Dazu gehören u. a. die Globalisierung und die zunehmende Subjektivierung der Arbeit [13], die Digitalisierung von Arbeitsprozessen, die mit einer Beschleunigung von Informations- und Arbeitsprozessen [14] und einer steigenden Menge an zu verarbeitenden Informationen einhergeht [15], betriebliche Reorganisationsprozesse und Rationalisierungsstrategien [16], neue Steuerungsformen [17], aber auch der Fachkräftemangel [18], von dem einige Branchen besonders stark betroffen sind. Alle diese Faktoren haben einen Einfluss darauf, wie viel Arbeit, mit welchen qualitativen Anforderungen in welcher Zeit von Beschäftigten erledigt werden muss, wie hoch also die Arbeitsintensität für die Beschäftigten ausfällt.

Mit dem Fortschreiten dieser Entwicklungen ist generell eine weitere Zunahme der Arbeitsintensität zu erwarten. Jedoch zeigen erste Ergebnisse der BIBB/BAuA-Erwerbstätigenbefragung [19], dass dies nicht für alle Facetten von Arbeitsintensität gilt. Zwar muss ein steigender Anteil der Beschäftigten gleichzeitig verschiedene Aufgaben betreuen, doch geben immer weniger Personen an, unter starkem Termin- oder Leistungsdruck bzw. sehr schnell arbeiten zu müssen. Andere Arbeitsintensitätsaspekte stabilisieren sich. Aber: Lässt sich dieser Befund für alle Beschäftigtengruppen verallgemeinern? Die genannten gesellschaftlichen, technologischen und wirtschaftlichen Entwicklungen betreffen nicht alle beruflichen Tätigkeiten, Qualifikationsniveaus, Arbeitszeitmodelle und Geschlechter gleichermaßen [20], was dafürspricht, dass die Entwicklung der Arbeitsintensität in den letzten Jahren differenzierter beschrieben werden muss. Deshalb fragen wir hier: Wie hat sich die Arbeitsintensität – differenziert nach Geschlecht, Arbeitszeitmodell, Qualifikationsniveau und beruflicher Tätigkeit – in den Jahren 2012 bis 2024 entwickelt und wo steht sie heute?

## Methoden

Grundlage für die folgenden Analysen sind die querschnittlichen BIBB/BAuA-Erwerbstätigenbefragungen aus den Jahren 2012, 2018 und 2024. Diese ursprünglich vom Bundesinstitut für Berufsbildung (BIBB) und dem Institut für Arbeitsmarkt- und Berufsforschung (IAB) gestartete Befragung wird seit dem Jahr 1979 regelmäßig durchgeführt. Die Bundesanstalt für Arbeitsschutz und Arbeitsmedizin (BAuA) ist seit 1998/1999 an der Befragung beteiligt. Nach dem Ausstieg des IAB wurde die ehemalige BIBB/IAB-Erhebung ab 2005/2006 zur BIBB/BAuA-Erwerbstätigenbefragung. Ziel dieser Befragung ist die Beschreibung der sich kontinuierlich verändernden Arbeitswelt. Dabei stehen Fragen zum Arbeitsplatz (Tätigkeitsschwerpunkte, Anforderungsniveau, Kenntnisanforderungen, Arbeitsanforderungen, Weiterbildungsbedarf, Arbeitsbedingungen, Arbeitsbelastungen usw.) und Fragen zu Beanspruchung und gesundheitlichen Beschwerden im Fokus.

Die Daten der 3 hier zugrunde gelegten Befragungen wurden jeweils mittels einer telefonischen, computerunterstützten Befragung bei rund 20.000 Erwerbstätigen in Deutschland erhoben. Seit 2018 erfolgt diese Telefonerhebung anhand einer Dual-Frame-Stichprobe, d. h., neben Interviews über Festnetznummern werden auch Interviews über Mobilfunknummern realisiert, um zu berücksichtigen, dass Menschen zum Teil keinen Festnetzanschluss mehr besitzen oder nutzen [21].

In die Befragung 2024 wurden – wie in die Vorgängerbefragungen – erwerbstätige Personen ab 15 Jahren mit einer bezahlten Tätigkeit von mindestens 10 h pro Woche einbezogen – inklusive mithelfenden Familienangehörigen und Personen mit maximal 3‑monatiger Unterbrechung der Tätigkeit (*N* 2012 = 20.036; *N* 2018 = 20.012; *N* 2024 = 20.006). Nichtdeutsche Staatsangehörige wurden nur bei hinreichenden Deutschkenntnissen befragt, ehrenamtlich tätige Personen wurden genauso ausgeschlossen wie Personen in einem Beschäftigungsverhältnis im Rahmen der ersten Ausbildung. In den folgenden Analysen werden aus diesen Stichproben die jeweils abhängig Beschäftigten betrachtet (*n* 2012 = 17.562; *n* 2018 = 17.852; *n* 2024 = 18.255). Selbstständige, freiberuflich Tätige, freie Mitarbeiter und mithelfende Familienangehörige sind damit nicht eingeschlossen. Die Merkmale der Stichproben zu den 3 Messzeitpunkten können der Tab. 1 entnommen werden.

**Tab. 1:** Stichprobenmerkmale der 3 BIBB/BAuA-Erwerbstätigenbefragungen 2012, 2018 und 2024

Merkmal	Ausprägung	BIBB/BAuA-Erwerbstätigenbefragung Angaben in %^a^		
		2012*, N* = 17.562^b^	2018*, N* = 17.852^b^	2024*, N* = 18.255^b^
Stellung im Beruf	Arbeiter	29	20	12
	Angestellte	64	74	82
	Beamte	6	6	6
Höchster Schulabschluss	Volks‑/Hauptschule/qual. Hauptschule/POS 8. Klasse	30	25	20
	Mittlere Reife/POS 10. Klasse	36	36	33
	Fachhochschulreife, Abitur, EOS	32	38	46
	Sonderschule/sonstige/ohne Abschluss	1	1	1
Staatsangehörigkeit	Deutsch	92	89	86
	Nichtdeutsch	8	11	14
Geschlecht	Männlich	54	53	53
	Weiblich	46	47	47
Alter	15–24	6	6	4
	25–34	21	22	22
	35–44	25	22	25
	45–54	31	29	21
	55–64	15	21	25
	65+	1	1	2
Arbeitszeit	Teilzeit	23	26	27
	Vollzeit	77	74	73
Anforderungsniveau	Helfer und Anlerntätigkeiten	9	8	5
	Fachlich ausgerichtete Tätigkeiten	58	56	50
	Komplexe Spezialistentätigkeiten	15	16	18
	Hochkomplexe Tätigkeiten	17	20	27
Berufe	Land‑, Forst- und Gartenbauberufe	1	2	2
	Fertigungsberufe	9	7	4
	Fertigungstechnische Berufe	14	12	12
	Bau- und Ausbauberufe	6	6	5
	Tourismus‑/Hotel‑/Gaststättenberufe	2	2	2
	Lebensmittelherstellung/-verarbeitung	3	3	2
	Medizinische Gesundheitsberufe	6	7	7
	Nichtmedizinische Gesundheitsberufe^c^	2	3	2
	Erziehung, soziale/hauswirtschaftliche Berufe, Theologie	4	6	6
	Lehrende/ausbildende Berufe	5	6	6
	Geistes‑/Gesellschafts‑/Wirtschaftswiss.; darstellende, unterhaltende Berufe	1	1	1
	Handelsberufe	9	8	8
	Berufe in Unternehmensführung/-organisation	8	9	10
	Unternehmensbezogene Dienstleistungsberufe	12	11	15
	IT-/naturwissenschaftliche Dienstleistungsberufe	4	5	8
	Sicherheitsberufe	2	3	2
	Verkehrs‑/Logistikberufe	9	10	8
	Reinigungsberufe	2	2	1

^a^Gewichtete Prozente^; b^gewichtete Anzahl; ^c^inkl. Körperpflege- und Wellnessberufe, Medizintechnik

*BAuA* Bundesanstalt für Arbeitsschutz und Arbeitsmedizin, *BIBB* Bundesinstitut für Berufsbildung, *EOS* Erweiterte Oberschule, *POS* Polytechnische Oberschule

Nach Abschluss der Erhebungen erfolgte jeweils ein Vergleich der Stichprobe mit Referenzstatistiken aus der Grundgesamtheit (Mikrozensen). Dadurch sollten die Stichproben in wesentlichen Merkmalen an Mikrozensusdaten angepasst werden, um die Repräsentativität für diese Merkmale zu gewährleisten. Die Anpassung der Ausgangsstichprobe an eine sogenannte Sollstruktur erfolgte für die Merkmale Geschlecht, Familienstand, Haushaltsgröße, Deutsch/Nichtdeutsch, Alter, Stellung im Beruf, höchster Schulabschluss und Bundesland des Wohnortes sowie West/Ost (Min./Max.-Gewichtungen 2012: 0,196–4,728; 2018: 0,200–4,898; 2024: 0,079–7,818). Im Wortlaut aufgenommene Berufsangaben wurden anschließend nach unterschiedlichen Schlüsseln (z. B. der Klassifikation der Wirtschaftszweige des Statistischen Bundesamts WZ 2008) kodiert, um Verbindungen zu anderen Datenquellen (z. B. EU-Daten) zu ermöglichen. Weitere Einzelheiten zu den Befragungen 2012, 2018 und 2024, insbesondere auch zu den vorgenommenen Gewichtungen, können den entsprechenden Methodenberichten entnommen werden [22–24].

Die Fragebögen der 3 Messzeitpunkte sind nicht zu 100 % identisch. Sie werden jeweils an Entwicklungen in der Arbeits- und Berufswelt in kleinen Teilen angepasst. Die Durchführung des aus rund 380 Fragen bestehenden Fragebogeninterviews dauerte im Durchschnitt gut 40 min. Für die Analyse der Arbeitsintensität wurden 3 über alle Messzeitpunkte hinweg gleich lautende Items ausgewählt (Eigenentwicklung), bei denen Daten zu allen 3 Messzeitpunkten vorlagen. Sie lauten: „Wie oft kommt es bei Ihrer Arbeit vor, …

1. … dass Sie verschiedenartige Arbeiten oder Vorgänge gleichzeitig im Auge behalten müssen?“
2. … dass Sie unter starkem Termin- oder Leistungsdruck arbeiten müssen?“
3. … dass Sie bis an die Grenzen Ihrer Leistungsfähigkeit gehen müssen?“

Die Antwortskala besteht aus den Möglichkeiten 1: häufig, 2: manchmal, 3: selten und 4: nie.

Berichtet wird jeweils der gewichtete prozentuale Anteil der Beschäftigten, der bei den einzelnen Items von häufigem Auftreten berichtet hat, unter Angabe der gewichteten 95 %-Konfidenzintervalle und der ungewichteten Anzahl. Neben den Angaben für die abhängig Beschäftigten gesamt zu den 3 Messzeitpunkten werden die Items auch nach verschiedenen demografischen und berufsbezogenen Merkmalen stratifiziert betrachtet.

## Ergebnisse

Das häufige Auftreten von *verschiedenartigen Arbeiten oder Vorgänge gleichzeitig im Auge behalten müssen *(im Folgenden auch „Multitasking“ genannt) wird im Jahr 2024 von 64,0 % der abhängig Beschäftigten berichtet (Tab. 2) und ist damit das am weitesten verbreitete der hier dargestellten Arbeitsintensitätsmerkmale. Im Vergleich zu 2018 ist es signifikant um +3,7 Prozentpunkte (%*P*), im Vergleich zu 2012 signifikant um insgesamt +5,6 %*P* gestiegen.

**Tab. 2:** Arbeitsintensität gesamt bei abhängig Beschäftigten 2012, 2018 und 2024 (Anteil häufig in %)

	2012			2018			2024		
	*N*^a^	Anteil häufig (%)^b^	95 %-KI^b^	*N*^a^	Anteil häufig (%)^b^	95 %-KI^b^	*N*^a^	Anteil häufig (%)^b^	95 %-KI^b^
Verschiedenartige Arbeiten gleichzeitig	11.013	58,4	57,5–59,4	11.773	60,3*	59,3–61,3	11.901	64,0***	62,7–65,2
Starker Termin- oder Leistungsdruck	9508	51,6	50,7–52,6	9130	47,7***	46,6–48,7	8044	43,0***	41,7–44,3
Arbeiten an Grenze der Leistungsfähigkeit	3070	16,3	15,6–17,0	2991	16,0 n. s.	15,3–16,8	2512	14,8***	13,9–15,8

^a^Ungewichtetes *n*; ^b^gewichtete Prozente

*p < 0,05, **p < 0,01, ***p < 0,001, n. s. = nicht signifikant (bzgl. Veränderungen zum vorherigen Messzeitpunkt); Signifikanz beruht auf dem korrigierten F nach Rao Scott (Variante der korrigierten Chi-Quadrat-Statistik zweiter Ordnung) sowie seinen Freiheitsgraden F

Anders beim *starken Termin- oder Leistungsdruck*: Von 51,6 % in 2012 über 47,7 % in 2018 hat sich der Anteil der Beschäftigten, die das mit häufig angeben, signifikant um insgesamt −8,6 %*P* auf 43,0 % reduziert. Das häufige *Arbeiten an der Grenze der Leistungsfähigkeit* ist zwischen 2012 mit 16,3 % und 2018 mit 16,0 % ungefähr gleichgeblieben und zeigt 2024 mit einem Wert von 14,8 % einen signifikanten Rückgang um −1,2 %*P* im Vergleich zu 2018 bzw. um −1,5 %*P* im Vergleich zu 2012.

Bei genauerem Hinsehen zeigt sich jedoch, dass die für die Gesamtstichprobe festgestellten Zu- und Abnahmen nicht für alle Beschäftigten gleichermaßen gelten.

### Geschlecht.

**Abb. 1:**
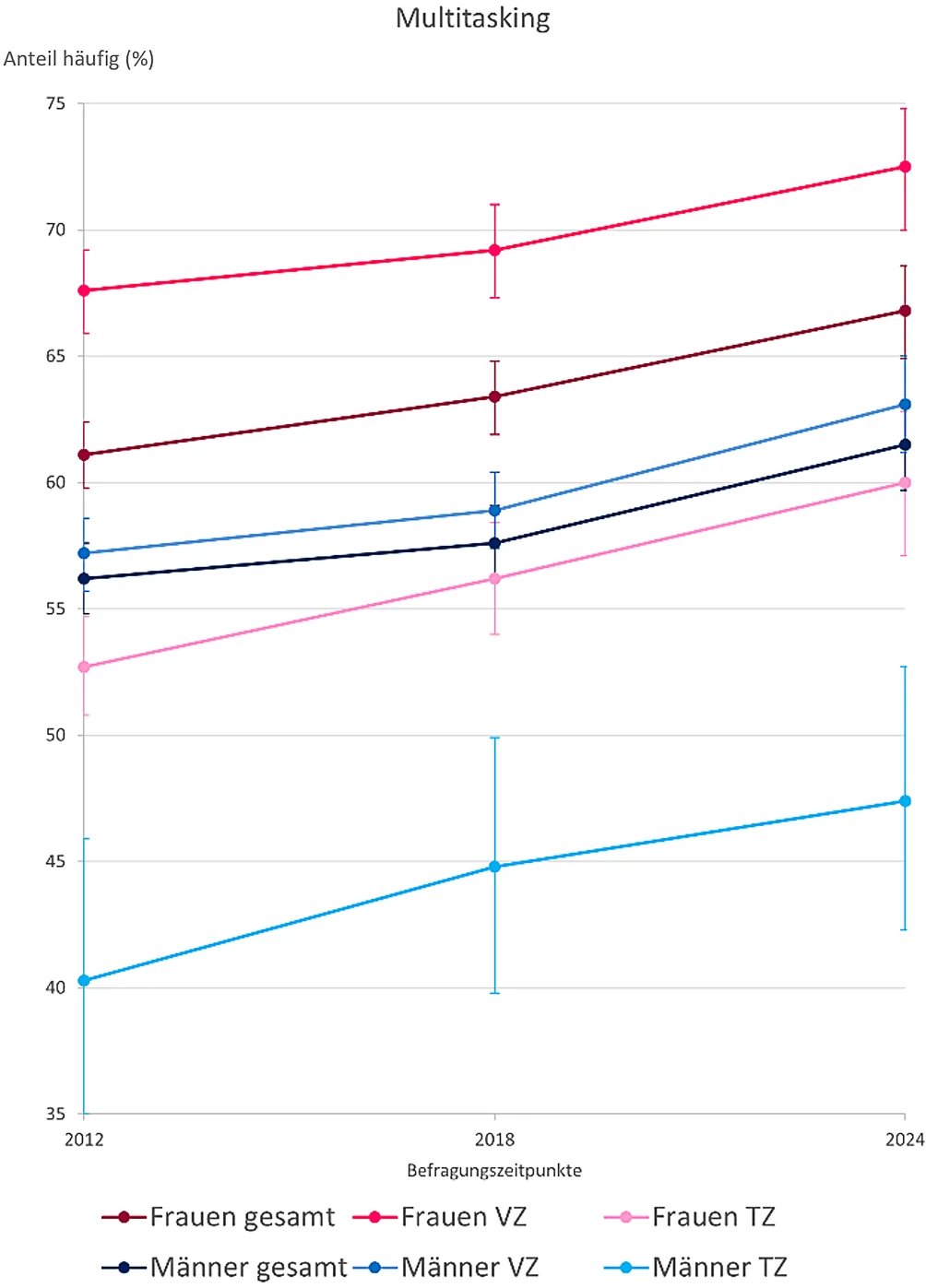
Multitasking nach Geschlecht und Arbeitszeit bei abhängig Beschäftigten 2012, 2018 und 2024 (Anteil häufig in %). *TZ* Teilzeit; *VZ* Vollzeit

**Abb. 2:**
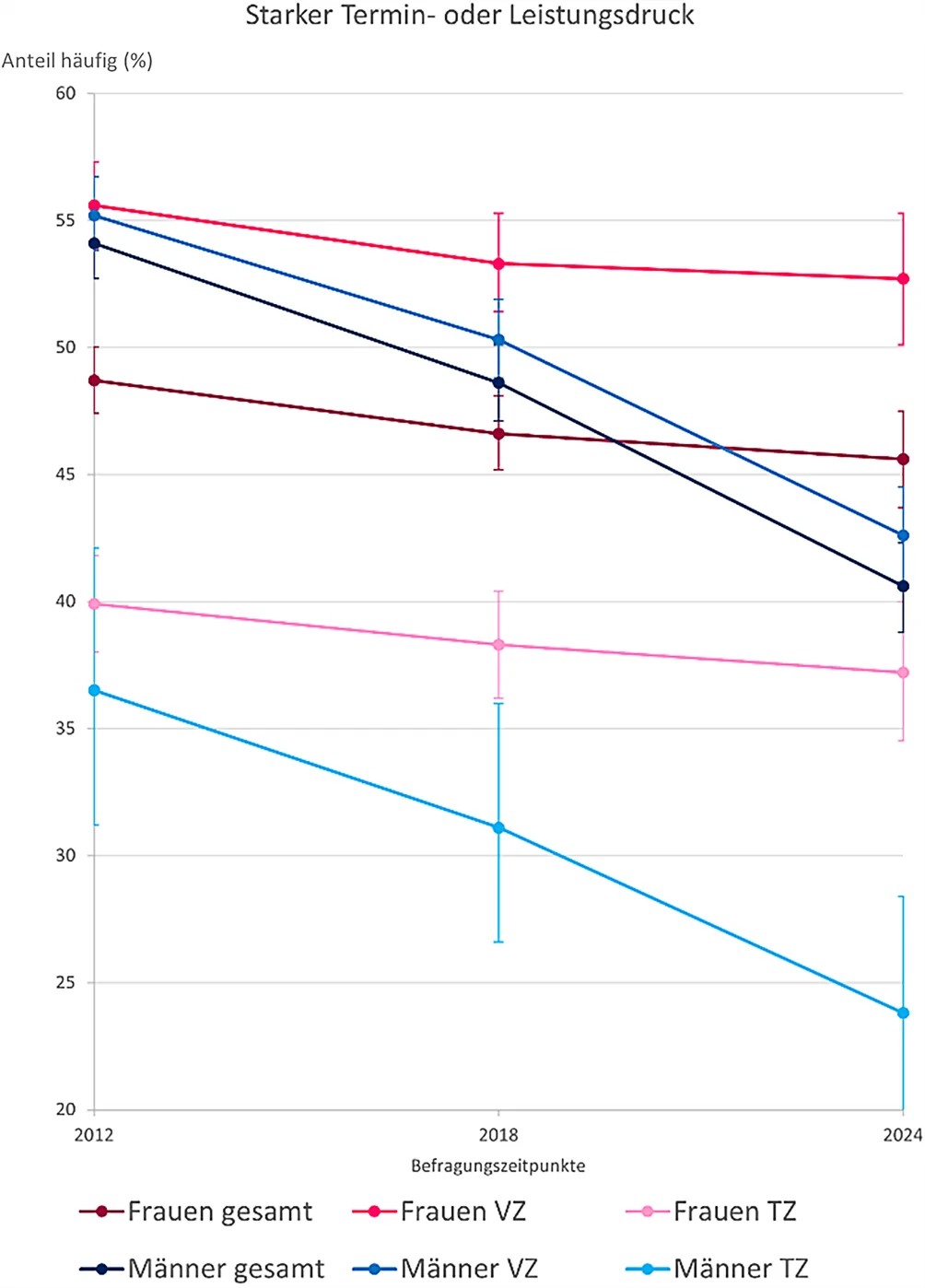
Starker Termin- oder Leistungsdruck nach Geschlecht und Arbeitszeit bei abhängig Beschäftigten 2012, 2018 und 2024 (Anteil häufig in %). *TZ* Teilzeit; *VZ* Vollzeit

**Abb. 3:**
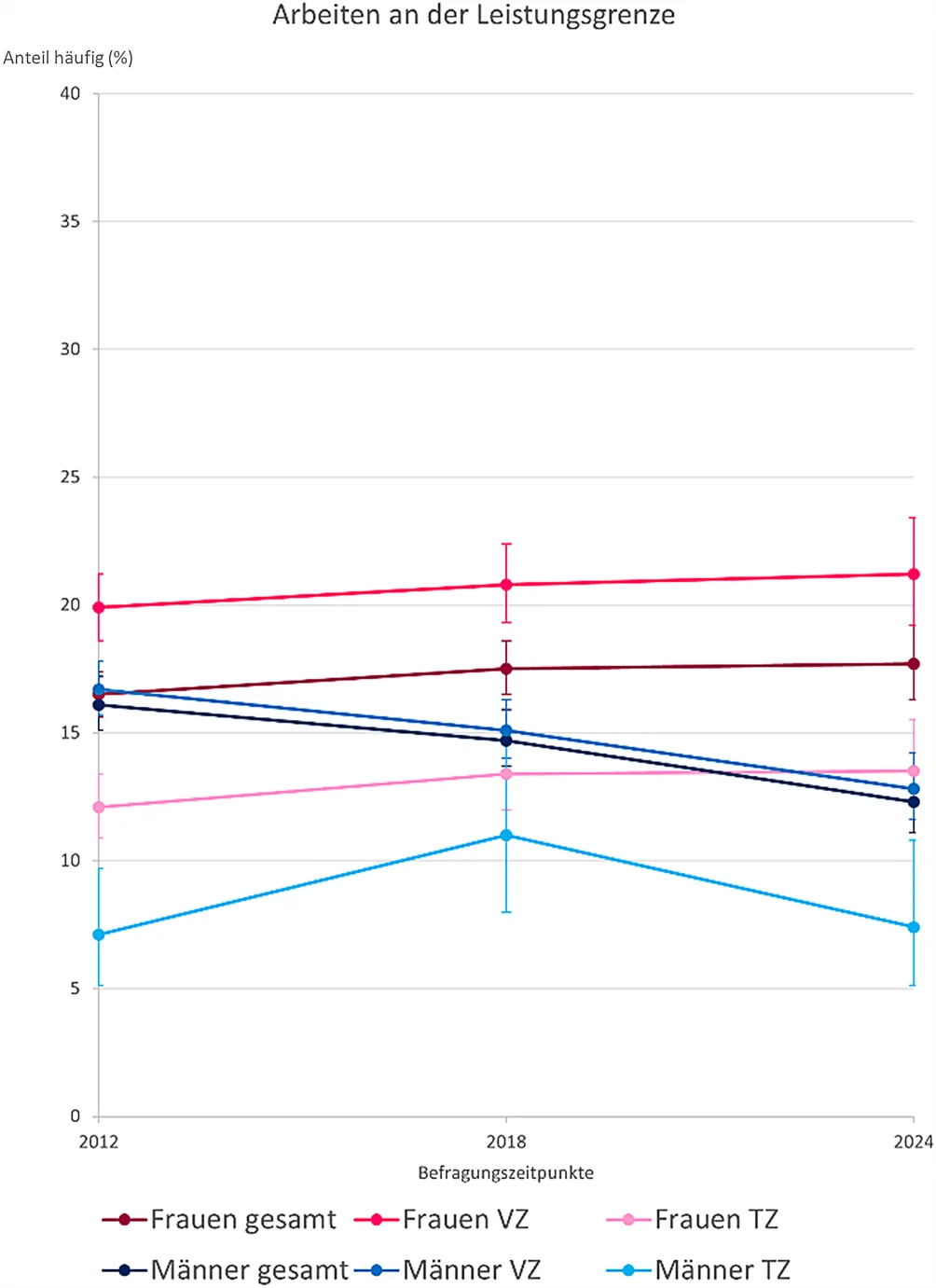
Arbeiten an der Leistungsgrenze nach Geschlecht und Arbeitszeit bei abhängig Beschäftigten 2012, 2018, und 2024 (Anteil häufig in %). *TZ* Teilzeit; *VZ* Vollzeit

So sind beispielsweise im Geschlechtervergleich signifikante Veränderungen häufiger und in höherem Maße bei Männern als bei Frauen festzustellen. Im Einzelnen sind signifikante Änderungen festzustellen in/bei (Abb. 1, 2, 3; vgl. zugrunde liegende Tabelle im Onlinematerial):

Multitasking

- 2018/2024 Männer +3,9 %*P*, Frauen +3,4 %*P*
- 2012/2018 Frauen +2,3 %*P*

Starker Termin- oder Leistungsdruck

- 2018/2024 Männer −8,0 %*P*
- 2012/2018 Männer −5,5 %*P*; Frauen −2,1 %*P*

Arbeiten an der Grenze der Leistungsfähigkeit

- 2018/2024 Männer −2,4 %*P*
- 2012/2018 Männer −1,4 %*P*

Zudem treten im Jahr 2024 alle 3 Arbeitsintensitätsmerkmale bei Frauen häufiger auf als bei Männern. So berichten 66,8 % der Frauen im Jahr 2024 von häufigem Multitasking, bei Männern sind dies 61,5 % (Abb. 1, 2, 3; vgl. zugrunde liegende Tabelle im Onlinematerial). Beim starken Termin- oder Leistungsdruck sagen in 2024 45,6 % der Frauen, dass dieser häufig auftritt, im Vergleich zu 40,6 % der Männer. Das Arbeiten an der Grenze der Leistungsfähigkeit wird in 2024 von 17,7 % der Frauen mit „häufig“ angegeben, bei den Männern sind es 12,3 %.

Noch deutlicher wird dieses Bild, wenn man Männer und Frauen außerdem nach *Teil- und Vollzeittätigkeit* unterteilt. Signifikante Veränderungen zeigen sich in/bei:

Multitasking

- 2018/2024 Männer Vollzeit +4,2 %*P*, Frauen Vollzeit +3,3 %*P*
- 2012/2018 Frauen Teilzeit +3,5 %*P*

Starker Termin- oder Leistungsdruck

- 2018/2024 Männer Vollzeit −7,7 %*P*, Männer Teilzeit −7,3 %*P*
- 2012/2018 Männer Vollzeit −4,9 %*P*

Arbeiten an der Grenze der Leistungsfähigkeit

- 2018/2024 Männer Vollzeit −2,3 %*P*; Männer Teilzeit −3,6 %*P*
- 2012/2018 Männer Vollzeit −1,6 %*P*, Männer Teilzeit +3,9 %*P*

Während also Männer sowohl in Teil- als auch in Vollzeit teilweise erhebliche signifikante Abnahmen zu verzeichnen haben (außer Männer in Teilzeit in 2012/2018 bei Arbeiten an der Grenze der Leistungsfähigkeit), stagniert die Arbeitsintensität bei Frauen auf hohem Niveau. Insgesamt geben im Jahr 2024 Frauen häufiger Arbeitsintensitätsmerkmale an als Männer, sowohl in Teilzeit- als auch in Vollzeitbeschäftigung. Deutlich am höchsten ist der Anteil von Beschäftigten, die von häufiger Arbeitsintensität berichten, bei vollzeitbeschäftigten Frauen. Dabei ist Arbeitsintensität bei Vollzeitbeschäftigten insgesamt weiter verbreitet als bei Teilzeitbeschäftigten.

### Anforderungsniveau.

Bei Betrachtung des beruflichen Anforderungsniveaus zeigt sich, dass signifikante Veränderungen vor allem bei fachlich ausgerichteten Tätigkeiten auftreten. Signifikante Abnahmen in den Häufigkeitsanteilen sind zudem am meisten bei starkem Termin- oder Leistungsdruck zu beobachten. Im Einzelnen sind folgende bedeutsame Veränderungen zu konstatieren (Tab. 3):

**Tab. 3:** Arbeitsintensität nach Anforderungsniveau bei abhängig Beschäftigten 2012, 2018 und 2024 (Anteil häufig in %)

	Jahr	Helfer und Anlerntätigkeiten			Fachlich ausgerichtete Tätigkeiten			Komplexe Spezialistentätigkeiten			Hochkomplexe Tätigkeiten		
		*N*^a^	Anteil häufig (%)^b^	95 % KI^b^	*N*^a^	Anteil häufig (%)^b^	95 % KI^b^	*N*^a^	Anteil häufig (%)^b^	95 % KI^b^	*N*^a^	Anteil häufig (%)^b^	95 % KI^b^
Verschiedenartige Arbeiten gleichzeitig	2012	502	36,4	33,2–39,7	5662	54,4	53,1–55,7	2082	68,7	66,5–70,9	2744	74,8	72,9–76,6
	2018	384	35,9 n. s.	32,1–39,9	5139	56,5*	55,0–57,9	2444	69,7 n. s.	67,4–71,8	3769	73,7 n. s.	71,7–75,6
	2024	247	40,8 n. s.	34,8–47,0	4399	60,7***	58,8–62,6	2547	69,7 n. s.	66,9–72,3	4600	71,1 n. s.	68,6–73,5
Starker Termin- oder Leistungsdruck	2012	501	37,9	34,7–41,3	4989	49,6	48,3–50,9	1748	57,7	55,3–60,0	2255	60,8	58,7–62,9
	2018	376	34,8 n. s.	31,1–38,7	4042	45,5***	44,1–47,0	1906	53,3**	50,9–55,7	2780	54,6***	52,5–56,7
	2024	183	32,7 n. s.	27,1–38,9	3013	40,2***	38,3–42,1	1677	45,0***	42,1–48,0	3111	49,0***	46,5–51,5
Arbeiten an Grenze der Leistungsfähigkeit	2012	217	15,4	13,1–17,9	1729	16,8	15,8–17,8	481	15,6	13,9–17,3	640	16,0	14,6–17,5
	2018	175	17,5 n. s.	14,6–20,9	1489	16,8 n. s.	15,7–17,9	516	13,8 n. s.	12,3–15,5	802	15,2 n. s.	13,8–16,8
	2024	101	15,4 n. s.	11,7–20,0	1087	15,3*	14,0–16,8	436	12,5 n. s.	10,7–14,6	869	15,2 n. s.	13,5–17,1

^a^Ungewichtetes *n*; ^b^gewichtete Prozente

*p < 0,05, **p < 0,01, ***p < 0,001, n. s. = nicht signifikant (bzgl. Veränderungen zum vorherigen Messzeitpunkt); Signifikanz beruht auf dem korrigierten F nach Rao Scott (Variante der korrigierten Chi-Quadrat-Statistik zweiter Ordnung) sowie seinen Freiheitsgraden F

Multitasking

- 2018/2024 fachlich ausgerichtete Tätigkeiten +4,2 %*P*
- 2012/2018 fachlich ausgerichtete Tätigkeiten +2,1 %*P*

Starker Termin- oder Leistungsdruck

- 2018/2024 fachlich ausgerichtete Tätigkeiten −5,3 %*P*, komplexe Spezialistentätigkeiten −8,3 %*P*, hochkomplexe Tätigkeiten −5,6 %*P*
- 2012/2018 fachlich ausgerichtete Tätigkeiten −4,1 %*P*, komplexe Spezialistentätigkeiten −4,4 %*P*, hochkomplexe Tätigkeiten −6,2 %*P*

Arbeiten an der Grenze der Leistungsfähigkeit

- 2018/2024 fachlich ausgerichtete Tätigkeiten −1,5 %*P*

Insgesamt, über alle Anforderungsniveaus hinweg betrachtet, steigt die Verbreitung der beiden Merkmale Multitasking und starker Termin- oder Leistungsdruck mit zunehmendem Anforderungsniveau, während das Arbeiten an der Grenze der Leistungsfähigkeit – abgesehen von der Gruppe mit komplexen Spezialistentätigkeiten – ungefähr gleichermaßen häufig auftritt. Am weitesten verbreitet sind Arbeitsintensitätsmerkmale, abgesehen vom Arbeiten an der Grenze der Leistungsfähigkeit, in der Gruppe der hochkomplexen Tätigkeiten.

### Berufsgruppen.

Bei der vergleichenden Betrachtung der Berufe im Hinblick auf die Entwicklungen der Arbeitsintensität zeigt sich ein gemischtes Bild (Tab. 4). Neben den Berufen, die tendenziell den Trends der Gesamtstichprobe folgen, also Zunahme beim Multitasking und Abnahme bei den beiden anderen Arbeitsintensitätsmerkmalen, gibt es Berufe in denen die Verbreitung der Arbeitsintensität im Großen und Ganzen stabil, aber auf hohem Niveau bleibt, wie z. B. bei den medizinischen Gesundheitsberufen. Darüber hinaus gibt es aber auch Berufsgruppen, in denen sich Tendenzen entgegen dem Gesamttrend abzeichnen, wie z. B. beim Multitasking in der Gruppe der „geistes-, gesellschafts- und wirtschaftswissenschaftlichen/darstellenden, unterhaltenden Berufe“. Darüber hinaus gibt es Berufe, bei denen sich über die 3 Erhebungszeitpunkte keine eindeutigen Tendenzen zeigen, wie z. B. in der Gruppe „Erziehung, soziale und hauswirtschaftliche Berufe, Theologie“, in der sich der starke Termin- oder Leistungsdruck zwischen 2012 und 2018 stark signifikant verringert hat, in der Tendenz zuletzt in 2024 aber wieder zuzunehmen scheint (nicht signifikant), bei gleichzeitigem signifikanten Anstieg des Anteils derjenigen, die häufig an der Grenze der Leistungsfähigkeit arbeiten. Insgesamt ist die Höhe der prozentualen Anteile in der Antwortkategorie häufig bei den einzelnen Arbeitsintensitätskriterien über die verschiedenen Berufsgruppen hinweg betrachtet sehr unterschiedlich.

**Tab. 4:** Arbeitsintensität nach Berufsgruppen bei abhängig Beschäftigten 2012, 2018 und 2024 (Anteil häufig in %)

	Jahr	Verschiedenartige Arbeiten gleichzeitig			Starker Termin- oder Leistungsdruck			Arbeiten an Grenze der Leistungsfähigkeit		
		*N*^a^	Anteil häufig (%)^b^	95 % KI^b^	*N*^a^	Anteil häufig (%)^b^	95 % KI^b^	*N*^a^	Anteil häufig (%)^b^	95 % KI^b^
Land‑, Forst- und Gartenbauberufe	2012	97	46,1	37,6–54,8	81	39,0	30,9–47,8	39	20,0	13,8–28,1
	2018	107	50,3 n. s.	41,1–59,5	76	37,8 n. s.	29,2–47,1	28	14,8 n. s.	9,2–23,0
	2024	124	55,8 n. s.	45,0–66,0	57	17,7**	11,4–26,5	30	7,6*	4,8–11,8
Fertigungsberufe	2012	501	48,4	44,6–52,1	538	52,9	49,2–56,7	174	16,5	13,9–19,4
	2018	407	50,3 n. s.	45,7–54,9	381	47,6 n. s.	43,0–52,3	97	12,2*	9,5–15,4
	2024	250	53,5 n. s.	46,5–60,3	207	40,7 n. s.	34,0–47,6	58	15,1 n. s.	10,3–21,6
Fertigungstechnische Berufe	2012	1126	52,7	49,9–55,5	1137	54,6	51,8–57,3	297	14,0	12,3–16,0
	2018	1063	51,0 n. s.	47,8–54,1	939	49,9*	46,7–53,0	227	11,6 n. s.	9,7–13,7
	2024	1171	57,3*	53,3–61,1	847	40,7***	37,0–44,6	185	9,3 n. s.	7,3–11,7
Bau- und Ausbauberufe	2012	414	49,0	44,6–53,4	427	54,6	50,2–58,9	148	20,9	17,5–24,9
	2018	427	51,3 n. s.	46,5–56,0	393	48,3 n. s.	43,6–53,0	119	15,4 n. s.	12,2–19,1
	2024	438	62,8***	56,5–68,7	310	41,7 n. s.	35,6–48,1	83	13,9 n. s.	10,0–19,1
Tourismus‑, Hotel- und Gaststättenberufe	2012	216	67,6	60,3–74,1	155	45,6	38,6–52,8	55	16,1	11,9–21,5
	2018	191	69,0 n. s.	60,7–76,2	134	48,2 n. s.	40,1–56,4	44	16,3 n. s.	11,0–23,4
	2024	147	74,2 n. s.	63,0–83,0	85	35,7 n. s.	26,0–46,7	30	18,9 n. s.	11,2–30,1
Lebensmittelherstellung u. -verarbeitung	2012	232	63,3	56,7–69,5	210	51,6	45,0–58,2	67	18,3	13,8–24,0
	2018	217	65,6 n. s.	58,4–72,1	166	49,5 n. s.	42,4–56,6	71	19,5 n. s.	14,7–25,2
	2024	117	69,3 n. s.	57,8–78,8	84	49,0 n. s.	37,7–60,3	31	20,5 n. s.	12,6–31,5
Medizinische Gesundheitsberufe	2012	954	68,1	64,8–71,2	881	63,8	60,4–67,0	324	22,4	19,7–25,3
	2018	1018	69,0 n. s.	65,4–72,4	923	63,0 n. s.	59,3–66,6	391	25,3 n. s.	22,3–28,5
	2024	846	68,2 n. s.	63,3–72,7	733	59,8 n. s.	54,9–64,5	275	21,4 n. s.	17,8–25,6
Nichtmedizinische Gesundheitsberufe^c^	2012	312	64,4	58,5–69,8	290	60,9	55,0–66,5	123	24,8	20,1–30,2
	2018	290	62,9 n. s.	56,6–68,9	262	60,7 n. s.	54,5–66,6	121	24,1 n. s.	19,4–29,6
	2024	202	68,4 n. s.	59,4–76,2	168	60,5 n. s.	51,5–68,8	81	27,4 n. s.	19,6–36,9
Erziehung, soziale, hauswirtschaftliche Berufe, Theologie	2012	690	73,0	69,0–76,7	392	39,8	35,9–43,9	206	18,7	15,9–21,9
	2018	846	71,5 n. s.	67,4–75,2	418	31,4**	27,7–35,4	215	17,4 n. s.	14,5–20,7
	2024	774	74,2 n. s.	69,1–78,7	398	36,7 n. s.	31,7–41,9	224	22,2*	18,1–26,9
Lehrende und ausbildende Berufe	2012	811	73,1	69,6–76,4	599	51,3	47,6–55,0	205	16,8	14,3–19,5
	2018	1191	71,0 n. s.	67,2–74,5	826	49,8 n. s.	46,1–53,4	298	16,6 n. s.	14,3–19,2
	2024	1099	70,9 n. s.	65,6–75,7	704	48,4 n. s.	43,3–53,5	289	19,4 n. s.	15,8–23,6
Wissenschaftliche Berufe; darstellende Berufe^d^	2012	85	71,1	59,4–80,6	73	65,4	54,5–74,8	32	24,7	16,4–35,4
	2018	128	71,4 n. s.	61,1–79,9	90	49,5*	39,3–59,6	23	15,7 n. s.	9,3–25,3
	2024	98	52,2*	37,1–66,9	68	59,4 n. s.	44,9–72,4	18	18,7 n. s.	8,6–36,1
Handelsberufe	2012	970	58,9	55,7–62,0	748	43,9	40,9–47,1	242	14,1	12,1–16,3
	2018	903	64,0*	60,3–67,6	649	43,7 n. s.	40,1–47,4	241	17,7 n. s.	14,9–20,9
	2024	810	64,3 n. s.	59,5–68,9	537	41,8 n. s.	37,1–46,6	173	16,4 n. s.	13,1–20,5
Berufe in Unternehmensführung und -organisation	2012	1174	68,3	65,4–71,1	924	52,9	49,8–55,9	252	13,4	11,4–15,6
	2018	1433	68,2 n. s.	65,1–71,1	1042	47,6*	44,5–50,6	269	12,0 n. s.	10,2–14,0
	2024	1531	70,5 n. s.	66,8–73,9	980	43,0 n. s.	39,4–46,8	271	13,3 n. s.	10,9–16,0
Unternehmensbezogene Dienstleistungsberufe	2012	1838	64,6	62,3–66,8	1554	53,5	51,2–55,8	409	12,8	11,4–14,3
	2018	1776	67,5 n. s.	64,9–70,0	1366	49,1*	46,5–51,8	378	14,2 n. s.	12,4–16,3
	2024	2215	67,4 n. s.	64,3–70,4	1506	47,6 n. s.	44,4–50,8	395	12,9 n. s.	10,9–15,3
IT- und naturwissenschaftliche Dienstleistungsberufe	2012	559	66,8	62,3–71,0	439	54,6	50,0–59,1	105	11,8	9,2–15,1
	2018	730	64,9 n. s.	60,6–68,9	515	45,6**	41,3–49,9	96	9,6 n. s.	7,1–13,0
	2024	1017	63,3 n. s.	58,5–67,9	584	34,2***	29,8–38,9	131	8,0 n. s.	5,8–11,0
Sicherheitsberufe	2012	264	63,9	57,6–69,7	186	42,6	36,6–48,8	73	17,5	13,3–22,7
	2018	297	59,1 n. s.	52,3–65,7	191	35,7 n. s.	29,8–42,1	72	16,9 n. s.	12,4–22,6
	2024	345	62,8 n. s.	55,0–70,0	210	33,5 n. s.	27,1–40,5	57	9,8 n. s.	6,2–15,2
Verkehrs- und Logistikberufe	2012	588	44,6	41,2–48,1	675	51,5	48,1–55,0	245	19,7	17,0–22,7
	2018	634	46,4 n. s.	42,8–50,1	644	47,9 n. s.	44,3–51,6	244	20,1 n. s.	17,3–23,4
	2024	579	55,8**	50,8–60,6	477	41,0*	36,3–46,0	144	16,2 n. s.	12,8–20,3
Reinigungsberufe	2012	84	22,1	17,1–28,0	103	29,1	23,4–35,5	50	12,9	9,3–17,5
	2018	78	33,5*	26,0–41,9	89	36,3 n. s.	28,7–44,7	48	22,1*	15,7–30,1
	2024	30	24,8 n. s.	15,0–38,1	29	23,4 n. s.	13,7–36,9	18	11,1*	5,9–20,0

^a^Ungewichtetes *n*; ^b^gewichtete Prozente; ^c^inkl. Körperpflege- und Wellnessberufe, Medizintechnik; ^d^Geistes‑, Gesellschafts‑, Wirtschafts- sowie darstellende und unterhaltende Berufe

*p < 0,05, **p < 0,01, ***p < 0,001, n. s. = nicht signifikant (bzgl. Veränderungen zum vorherigen Messzeitpunkt); Signifikanz beruht auf dem korrigierten F nach Rao Scott (Variante der korrigierten Chi-Quadrat-Statistik zweiter Ordnung) sowie seinen Freiheitsgraden F

Im Jahr 2024 ist das häufige *Multitasking* mit 74,2 % in der Berufsgruppe „Erziehung, soziale und hauswirtschaftliche Berufe, Theologie“ sowie in „Tourismus‑, Hotel- und Gaststättenberufen“ am weitesten verbreitet. Die geringste Verbreitung weisen mit 24,8 % die Reinigungsberufe auf. Bei Betrachtung der Entwicklung zeigt sich für den Zeitraum 2012–2024 die größte Zunahme für Bau- und Ausbauberufe (+13,8 %*P*) sowie für Verkehrs- und Logistikberufe (+11,2 %*P*).

Bedeutsame Veränderungen bzgl. Multitaskings gibt es für folgende Zeitintervalle und Berufsgruppen:

- 2018/2024 Bau- und Ausbauberufe +11,5 %*P*, Verkehrs- und Logistikberufe +9,4 %*P*, fertigungstechnische Berufe +6,3 %*P* wissenschaftliche/darstellende Berufe −19,2 %*P,*
- 2012/2018 Reinigungsberufe +11,4 %*P*, Handelsberufe +5,1 %*P.*

Häufiger starker *Termin- oder Leistungsdruck* tritt im Jahr 2024 bei den nichtmedizinischen Gesundheitsberufen (60,5 %), den medizinischen Gesundheitsberufen (59,8 %) und den „geistes-, gesellschafts- und wirtschaftswissenschaftlichen/darstellenden, unterhaltenden Berufen“ (59,4 %) am häufigsten auf. Am geringsten sind die Anteile in der Antwortkategorie „häufig“ in den Land‑, Forst- und Gartenbauberufen (17,7 %) sowie in den Reinigungsberufen (23,4 %). Dabei sind die stärksten Abnahmen zwischen 2012 und 2024 in den Land‑, Forst- und Gartenbauberufen (−21,3 %*P*) und den IT- und naturwissenschaftlichen Dienstleistungsberufen (−20,4 %*P*) zu verzeichnen.

Im Einzelnen sind signifikante Veränderungen bzgl. des Termin- oder Leistungsdrucks für folgende Zeitintervalle und Berufsgruppen zu konstatieren:

- 2018/2024 Land‑, Forst- und Gartenbauberufe −20,1 %*P*, IT- und naturwissenschaftliche Dienstleistungsberufe −11,4 %*P*, fertigungstechnische Berufe −9,2 %*P*, Verkehrs- und Logistikberufe −6,9 %*P,*
- 2012/2018 wissenschaftliche/darstellende Berufe −15,9 %*P*, IT- und naturwissenschaftliche Dienstleistungsberufe −9,0 %*P*, Erziehung, soziale, hauswirtschaftliche Berufe, Theologie −8,4 %*P*, Berufe in Unternehmensführung und -organisation −5,3 %*P*, fertigungstechnische Berufe −4,7 %*P*, unternehmensbezogene Dienstleistungsberufe −4,4 %*P.*

Das häufige *Arbeiten an der Grenze der Leistungsfähigkeit* ist im Jahr 2024 in den nichtmedizinischen Gesundheitsberufen (27,4 %), in der Berufsgruppe „Erziehung, soziale und hauswirtschaftliche Berufe, Theologie“ (22,2 %) und in den medizinischen Gesundheitsberufen (21,4 %) am weitesten verbreitet. Am wenigsten Verbreitung findet dieses Kriterium in den Land‑, Forst- und Gartenbauberufen (7,6 %), den IT- und naturwissenschaftlichen Dienstleistungsberufen (8,0 %) und den fertigungstechnischen Berufen (9,3 %). Die stärkste Abnahme zeigt sich zwischen 2012 und 2024 in den Land‑, Forst- und Gartenbauberufen (−12,4 %*P*).

Im statistischen Sinne bedeutsame Veränderungen bzgl. Arbeitens an der Grenze der Leistungsfähigkeit sind in folgenden Zeiträumen und Berufsgruppen zu beobachten:

- 2018/2024 Reinigungsberufe −11,0 %*P*, Land‑, Forst- und Gartenbauberufe −7,2 %*P*, Erziehung, soziale, hauswirtschaftliche Berufe, Theologie +4,8 %*P,*
- 2012/2018 Fertigungsberufe −4,3 %*P*, Reinigungsberufe +9,2 %*P.*

Insgesamt sind nichtmedizinische und medizinische Gesundheitsberufe gefolgt von Berufen in Lebensmittelherstellung und -verarbeitung sowie lehrende und ausbildende Berufe am meisten vom häufigen Auftreten der hier untersuchten Arbeitsintensitätsmerkmale betroffen, am wenigsten Reinigungsberufe und die Land‑, Forst- und Gartenbauberufe.

## Diskussion

Zusammenfassend bleibt festzuhalten, dass für abhängig Beschäftigte insgesamt die Auftretenshäufigkeit von Arbeitsintensität im Merkmal Multitasking zu- und für starken Termin- oder Leistungsdruck sowie für das Arbeiten an der Grenze der Leistungsfähigkeit abgenommen hat. Dabei hält die Verbreitung von Arbeitsintensität weiter auf hohem Niveau an. Jedoch lassen sich diese Gesamtergebnisse nicht für alle Beschäftigtengruppen verallgemeinern. Sowohl Stand als auch Entwicklung der Arbeitsintensität unterscheiden sich je nach betrachteter Beschäftigtengruppe. Dies könnte damit zusammenhängen, dass die verschiedenen Beschäftigtengruppen unterschiedlich stark von gesellschaftlichen, technologischen und wirtschaftlichen Entwicklungen, die als Treiber hoher Arbeitsintensität diskutiert werden [13–18], betroffen sind.

### Arbeitsintensität bei Frauen.

Frauen (insbesondere in Vollzeitbeschäftigung) berichten nicht nur öfter von häufiger Arbeitsintensität, sondern sie ist auch bei ihnen im Vergleich zu 2018 (bezogen auf starken Termin- oder Leistungsdruck und das Arbeiten an der Grenze der Leistungsfähigkeit) relativ stabil geblieben – anders als bei männlichen Beschäftigten. Dies steht im Einklang mit Ergebnissen anderer Studien, die ebenfalls zeigen, dass Frauen häufiger hoher Arbeitsintensität ausgesetzt sind [25], und weist auf einen besonderen Gestaltungsbedarf für die Beschäftigtengruppe *vollzeitbeschäftigter Frauen* hin.

Über konkretere Gründe für diese Entwicklungen kann nur spekuliert werden. Möglicherweise sind Frauen eher in Berufen vollzeitbeschäftigt, in denen häufiger Termin- oder Leistungsdruck und Arbeit an der Grenze der Leistungsfähigkeit weitverbreitet ist. So ist der Anteil von vollzeitbeschäftigten Frauen sowohl in den Gesundheits- als auch in den Lehr- und Erziehungsberufen besonders hoch, also ausgerechnet in den Berufsgruppen, die durch eine hohe Arbeitsintensität gekennzeichnet sind.

Darüber hinaus zeigen Auswertungen des IAB [26], dass der Anteil von weiblichen Vollzeitbeschäftigten mit zunehmender Qualifikation ansteigt. Wie wir oben zeigen konnten, ist genau in der Gruppe mit der höchsten Qualifikation (hochkomplexe Tätigkeiten) die Arbeitsintensität am höchsten.

Des Weiteren leisten Frauen deutlich mehr Care-Arbeit als Männer, auch wenn sie vollzeitbeschäftigt sind [27]. Familiäre Verpflichtungen erschweren eine Extensivierung der Arbeit in Form von Mehrarbeit bzw. Überstunden. Somit kann steigenden quantitativen Anforderungen bei der Arbeit nur mit Intensivierung begegnet werden. Befunde, dass vollzeitbeschäftigte Frauen seltener überlange Arbeitszeiten (> 48 h/Wo) berichten (17 % Frauen vs. 30 % Männer; [28]), sprechen für diese Annahme.

### Anforderungsniveau und Arbeitsintensität.

Weiterhin zeigt sich, dass Merkmale hoher Arbeitsintensität mit steigendem Anforderungsniveau zunehmen und am häufigsten bei hochkomplexen Tätigkeiten auftreten. Die hohe Prävalenz bei hochkomplexen Tätigkeiten mag darin begründet liegen, dass Komplexität eine zentrale Dimension von Arbeitsintensität darstellt, demzufolge eine hohe Arbeitsintensität bei dieser Gruppe tätigkeitsimmanent ist. Der Befund, dass Arbeitsintensität mit steigender Qualifikation zunimmt, zeigt sich auch im Arbeitszeitreport (Beschäftigte mit hoher Arbeitsintensität: Basisarbeit 33 %, Facharbeit 43 %, hochqualifizierte Arbeit 50 %; [29]).

Zudem fallen auch Führungskräfte in diese Beschäftigtengruppe. Die Tätigkeit von Führungskräften ist durch eine hohe Arbeitsmenge, eine starke Gleichzeitigkeit und Fragmentierung der Arbeit sowie eine hohe Verantwortung für die eigene Arbeit und das Funktionieren eines Teams gekennzeichnet, was die besonders hohe Arbeitsintensität erklären kann [30].

### Verschiedene Berufsgruppen und Arbeitsintensität.

Im Hinblick auf die Berufsgruppen zeigen sich überdurchschnittliche Werte vor allem in medizinischen und nichtmedizinischen Gesundheitsberufen, in der Lebensmittelherstellung und -verarbeitung sowie in lehrenden und ausbildenden Berufen. Hier liegen die Prozentwerte der Antwortkategorien häufig bei allen 3 Kriterien über dem Durchschnitt. Vor allem in den Gesundheitsberufen, aber auch in lehrenden Berufen könnte der starke Fachkräftemangel ein zentraler Treiber hoher Arbeitsintensität sein [31]. Auch der hohe Frauenanteil in diesen Berufen könnte zu diesem Ergebnis beigetragen haben. Denn Frauen können – wie oben diskutiert – aufgrund von zu leistender Care-Arbeit ihre Arbeit vielfach nur intensivieren. Zusammenfassend bleibt festzuhalten, dass die Arbeitsintensität in vielen Berufen weiterhin relativ hoch ist und Entwicklungen in einzelnen Facetten der Arbeitsintensität und mehreren Berufsgruppen nicht immer dem Gesamttrend entsprechen.

### Maßnahmen zum Schutz vor zu hoher Arbeitsintensität.

Da Gefährdungen im Rahmen des Arbeitsschutzes im Grundsatz zunächst an der Quelle zu vermeiden sind, heißt dies zunächst, darauf zu achten, dass länger anhaltende Arbeitsintensität vermieden wird. Die in diesem Beitrag genannten Beschäftigtengruppen bei denen Arbeitsintensität überdurchschnittlich häufig auftritt, bieten einen ersten Anhaltspunkt für Schwerpunktsetzungen zur Entwicklung von Schutzmaßnahmen.

Betriebe sowie interne und externe Stakeholder können dabei auf erprobte Gestaltungsansätze zurückgreifen [3, 12, 32–34]. Ein Angebots- und Auftragsmanagement, das die zur Verfügung stehenden Ressourcen (z. B. Personal, Zeit) berücksichtigt, eine klare Begrenzung von Arbeitsaufgaben (z. B. durch konkrete Stellenbeschreibungen), eine bewusste Arbeitsverteilung unter Berücksichtigung individueller Qualifikationen und Präferenzen der Beschäftigten einerseits und der Schwierigkeit und Komplexität der Arbeitsaufgaben andererseits sind Wege, um Beschäftigte vor hoher Arbeitsintensität zu schützen [12].

Neben diesen Gestaltungsmaßnahmen, die hoher Arbeitsintensität entgegenwirken, sollte zudem ganz besonders auf ausreichende Ruhe- und Erholzeiten geachtet werden, um negativen Beanspruchungsfolgen vorzubeugen. Dabei gilt es nicht nur, Pausenausfall oder Pausenverkürzung innerhalb des Arbeitskontextes zu vermeiden, sondern auch Pausen rechtzeitig einzulegen und gut zu gestalten [35, 36]. Darüber hinaus sollte das *Abschalten von der Arbeit* (*Detachment*) außerhalb des Arbeitskontextes (im Feierabend, am Wochenende, im Urlaub) in den Blick genommen werden. Denn um langfristig gesund und beschäftigungsfähig zu bleiben, ist es wichtig, frühzeitig Strategien zur mentalen Erholung zu erlernen und diese regelmäßig in den Arbeitsalltag einzubeziehen [36]. Dies ist umso wichtiger bei Vollzeitbeschäftigten: Die rechtzeitige Planung, aber auch die Einhaltung von Pausenzeiten während der Arbeit sind dabei ebenso zentral wie die Begrenzung der täglichen und wöchentlichen Arbeitszeit sowie die Einhaltung der täglichen Ruhezeit [11, 12].

Ebenso sind in den medizinischen und nichtmedizinischen Gesundheitsberufen, in Lebensmittelberufen sowie in lehrenden und ausbildenden Berufen weitere Anstrengungen zur Vermeidung von Gefährdungen durch hohe Arbeitsintensität nötig – insbesondere da es sich hier z. T. um Berufsgruppen handelt, die von Fachkräftemangel geprägt sind. Daneben gilt es, im Analysezeitraum festgestellte besonders hohe Zunahmen (wie z. B. hinsichtlich des Multitaskings in Bauberufen und in Verkehrs- und Logistikberufen) bzw. Zunahmen entgegen den Trends (wie z. B. zwischen 2018 und 2024 für starken Termin- oder Leistungsdruck in der Gruppe Erziehung, soziale, hauswirtschaftliche Berufe, Theologie) weiter zu beobachten.

### Limitationen.

In diesem Beitrag werden Prävalenzen ausgewählter Arbeitsintensitätsaspekte auf Basis von Einzelitems berichtet, was das Konstrukt nicht in seiner ganzen Breite und Differenziertheit abbildet. Jedoch kann wegen der Themenfülle, der begrenzten Interviewzeit und der Erhebung per Telefon nicht auf umfassende Itembatterien zurückgegriffen werden. Allerdings wird in der neueren Literatur auf die potenzielle Güte von Single-Items verwiesen [37]. Darüber hinaus handelt es sich bei den Daten um Ergebnisse aus mehreren Querschnittsbefragungen, die keine Veränderungen der Arbeitsintensität auf individueller Ebene abbilden. Gleichwohl vermögen diese wiederholt erhobenen Querschnittsdaten Aussagen über Stand und Entwicklung von Sicherheit und Gesundheit bei der Arbeit auf Grundlage repräsentativer Stichproben für die jeweilige Erwerbsbevölkerung zu treffen. Die großen Stichprobenumfänge erlauben dabei differenzierte Analysen für verschiedene Beschäftigtengruppen. Über mögliche Treiber hoher Arbeitsintensität kann hier nur diskutiert werden, eine theorie- und hypothesengeleitete Überprüfung muss an anderer Stelle erfolgen.

## Fazit

Insgesamt ist festzuhalten, dass Stand und Entwicklung der Arbeitsintensität je nach betrachteter Facette und Beschäftigtengruppe unterschiedlich ausfallen und nicht immer dem Gesamttrend entsprechen. Entwarnung kann nicht gegeben werden, da – trotz einiger zu verzeichnender Rückgänge – die Arbeitsintensität in vielen Beschäftigtengruppen weiterhin relativ hoch ist. Daher muss sie als zentraler Schlüsselfaktor im Belastungs-Beanspruchungsgeschehen in der präventiven Arbeitsgestaltung und im Rahmen der gesetzlich vorgeschriebenen Gefährdungsbeurteilung berücksichtigt werden. Die Ergebnisse zeigen außerdem, dass weiterhin eine Auseinandersetzung mit hoher Arbeitsintensität im konkreten Tätigkeitskontext erforderlich ist. Dabei besteht allerdings noch Forschungsbedarf hinsichtlich der Frage, welche Veränderungen im Arbeitskontext die verschiedenen Ab- und Zunahmen der Arbeitsintensität in den jeweiligen Beschäftigtengruppen erklären können. Die hier diskutierten Vermutungen könnten hierfür erste Impulse bieten.

## Supplementary Information

Onlinematerial: Tabellen zu Abb. 1, Abb. 2 und Abb. 3

## Data Availability

Die analysierten Datenfiles können beim GESIS Leibniz-Institut für Sozialwissenschaften bezogen werden.
