# Supplementary material for: Work intensity—current situation and trends: Cross-sectional data comparison based on the 2012, 2018, and 2024 BIBB/BAuA Employment Surveys
Source: Bundesgesundheitsblatt Gesundheitsforschung Gesundheitsschutz. 2026 Sep 7;69(11):1351–62. [Article in German] doi: 10.1007/s00103-026-04291-x (PMC13714107; doi:10.1007/s00103-026-04291-x)
Supplement: Supplementary file 1 [file 103_2026_4291_MOESM1_ESM.pdf]

Onlinematerial: Tabellen zu Abb. 1, Abb. 2 und Abb. 3

Arbeitsintensität nach Geschlecht bei abhängig Beschäftigten 2012, 2018 und 2024 (Anteil häufig in %)

		Männer			Frauen		
	Jahr	n[a]	Anteil häufig (%)[b]	95% KI[b]	n[a]	Anteil häufig (%)[b]	95% KI[b]
Verschiedenartige Arbeiten gleichzeitig	2012	4.884	56,2	54,8-57,6	6.129	61,1	59,8-62,4
	2018	5.536	57,6 n.s.	56,1-59,1	6.237	63,4*	61,9-64,8
	2024	6.427	61,5***	59,7-63,2	5.474	66,8*	64,9-68,6
Starker Termin- oder Leistungsdruck	2012	4.553	54,1	52,7-55,5	4.955	48,7	47,4-50,0
	2018	4.472	48,6***	47,1-50,1	4.658	46,6*	45,2-48,1
	2024	4.344	40,6***	38,8-42,3	3.700	45,6 n.s.	43,7-47,5
Arbeiten an Grenze der Leistungsfähigkeit	2012	1.333	16,1	15,1-17,2	1.737	16,5	15,6-17,4
	2018	1.258	14,7*	13,7-15,9	1.733	17,5 n.s.	16,5-18,6
	2024	1.116	12,3***	11,1-13,5	1.396	17,7 n.s.	16,3-19,2

[a]ungewichtetes n; [b]gewichtete Prozente; *p < 0,05, **p < 0,01, ***p < 0,001, n.s. = nicht signifikant (bzgl. Veränderungen zum vorherigen Messzeitpunkt); Signifikanz beruht auf dem korrigierten F nach Rao Scott (Variante der korrigierten Chi-Quadrat-Statistik zweiter Ordnung) sowie seinen Freiheitsgraden F.

Arbeitsintensität nach Arbeitszeit und Geschlecht bei abhängig Beschäftigten 2012, 2018 und 2024 (Anteil häufig in %)

		Teilzeit (tatsächliche Arbeitszeit < 35 Std./Wo						Vollzeit (tatsächliche Arbeitszeit >= 35 Std./Wo					
		Männer			Frauen			Männer			Frauen		
	Jahr	n[a]	Anteil häufig (%)[b]	95% KI[b]	n[a]	Anteil häufig (%)[b]	95% KI[b]	n[a]	Anteil häufig (%)[b]	95% KI[b]	n[a]	Anteil häufig (%)[b]	95% KI[b]
Verschiedenartige Arbeiten gleichzeitig	2012	232	40,3	35,0-45,9	2.215	52,7	50,8-54,7	4.644	57,2	55,7-58,6	3.899	67,6	65,9-69,2
	2018	370	44,8 n.s.	39,8-49,9	2.431	56,2*	54,0-58,4	5.166	58,9 n.s.	57,4-60,4	3.806	69,2 n.s.	67,3-71,0
	2024	561	47,4 n.s.	42,3-52,7	2.112	60,0 n.s.	57,1-62,8	5.866	63,1***	61,2-65,0	3.362	72,5*	70,0-74,8
Starker Termin- oder Leistungsdruck	2012	192	36,5	31,2-42,1	1.668	39,9	38,0-41,8	4.355	55,2	53,8-56,7	3.272	55,6	53,9-57,3
	2018	242	31,1 n.s.	26,6-36,0	1.645	38,3 n.s.	36,2-40,4	4.230	50,3***	48,8-51,9	3.013	53,3 n.s.	51,4-55,3
	2024	303	23,8*	19,7-28,4	1.261	37,2 n.s.	34,5-40,0	4.041	42,6***	40,7-44,5	2.439	52,7 n.s.	50,1-55,3
Arbeiten an Grenze der Leistungsfähigkeit	2012	50	7,1	5,1-9,7	518	12,1	10,9-13,4	1.280	16,7	15,7-17,8	1.211	19,9	18,6-21,2
	2018	73	11,0*	8,0-14,9	541	13,4 n.s.	12,0-15,0	1.185	15,1*	14,0-16,3	1.192	20,8 n.s.	19,3-22,4
	2024	82	7,4*	5,1-10,8	430	13,5 n.s.	11,6-15,5	1.034	12,8**	11,6-14,2	966	21,2 n.s.	19,2-23,4

[a]ungewichtetes n; [b]gewichtete Prozente; *p < 0,05, **p < 0,01, ***p < 0,001, n.s. = nicht signifikant (bzgl. Veränderungen zum vorherigen Messzeitpunkt); Signifikanz beruht auf dem korrigierten F nach Rao Scott (Variante der korrigierten Chi-Quadrat-Statistik zweiter Ordnung) sowie seinen Freiheitsgraden F.
